# Supplementary material for: Using Electroporation to Improve and Accelerate Zebrafish Embryo Toxicity Testing
Source: Micromachines (Basel). 2023 Dec 26;15(1):49. doi: 10.3390/mi15010049 (PMC10819337; doi:10.3390/mi15010049)
Supplement: Supplementary file 1 [file micromachines-15-00049-s001.zip › micromachines-2755404-supplementary.pdf]

Supplementary Figures :

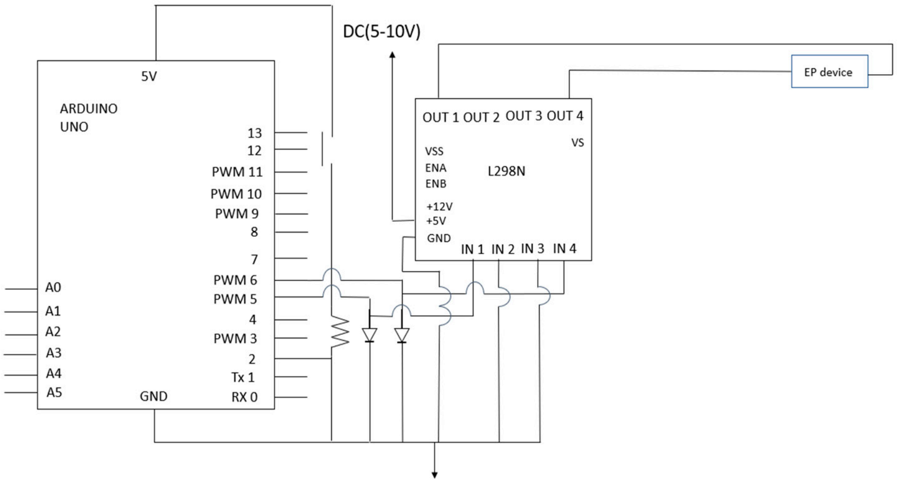

(a)

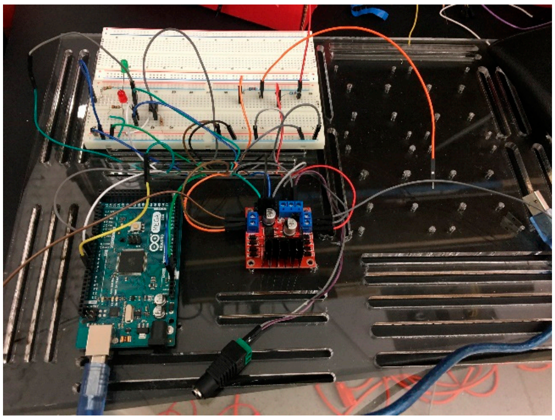

(b)

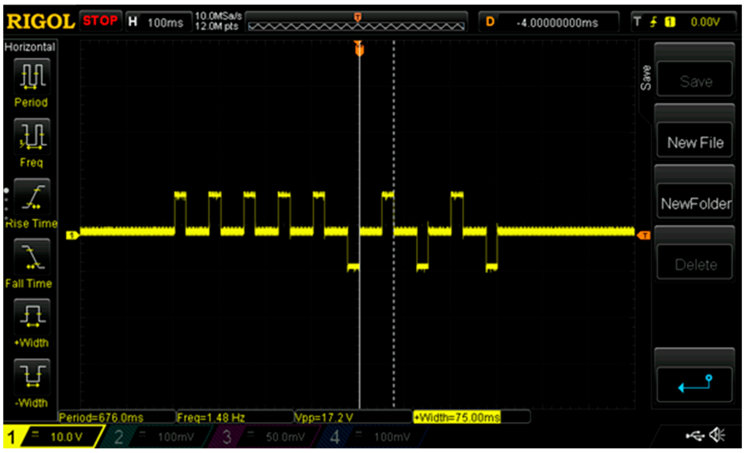

(c)

Supplementary Figure S1: (a) Electroporation controller schematic circuit diagram for voltage 5-25V (b) actual circuit (c) applied pulses

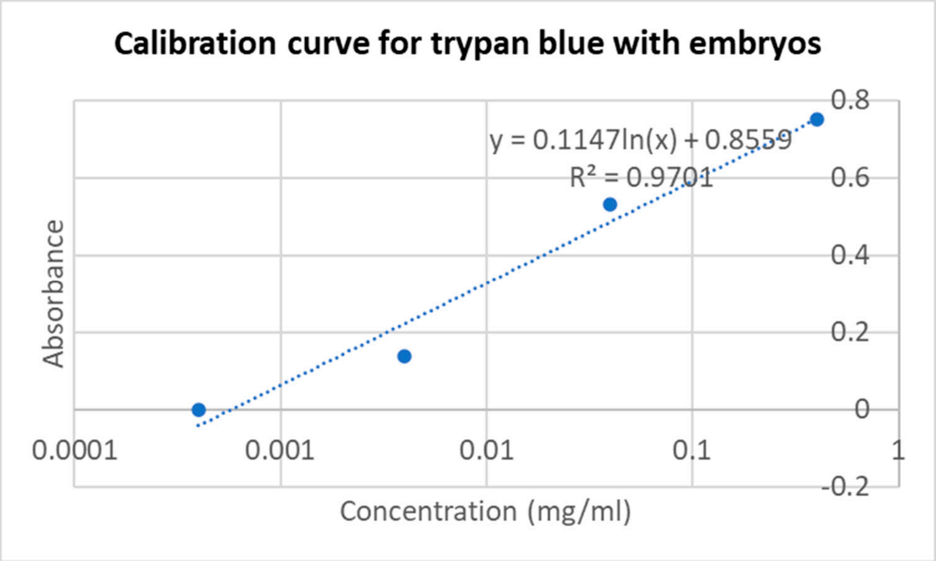

Supplementary Figure S2 : Calibration curve for trypan blue when embryos are soaked in various concentration of trypan blue for 5 hours. After that images are taken and RGB values are calculated to determine absorbance.

Ordered Differences Report

Level	- Level	Difference	Std Err Dif	Lower CL	Upper CL	p-Value
20V	No elec	4.827555	0.3308247	4.11285	5.542259	<.0001*
20V	5V	3.287706	0.3424361	2.54792	4.027495	<.0001*
20V	10V	3.187565	0.3308247	2.47286	3.902268	<.0001*
15V	No elec	3.121162	0.3308247	2.40646	3.835866	<.0001*
20V	15V	1.706393	0.3954112	0.85216	2.560627	0.0008*
10V	No elec	1.639990	0.2500800	1.09973	2.180255	<.0001*
15V	5V	1.581313	0.3424361	0.84153	2.321102	0.0005*
5V	No elec	1.539849	0.2652499	0.96681	2.112886	<.0001*
15V	10V	1.481172	0.3308247	0.76647	2.195875	0.0006*
10V	5V	0.100142	0.2652499	-0.47290	0.673179	0.7119

(a)

Ordered Differences Report

Level	- Level	Difference	Std Err Dif	Lower CL	Upper CL	p-Value
20V	No elec	0.2122619	0.0145987	0.180723	0.2438005	<.0001*
15V	No elec	0.1693751	0.0145987	0.137837	0.2009136	<.0001*
10V	No elec	0.1194420	0.0110356	0.095601	0.1432829	<.0001*
5V	No elec	0.1147348	0.0117050	0.089448	0.1400219	<.0001*
20V	5V	0.0975271	0.0151111	0.064882	0.1301726	<.0001*
20V	10V	0.0928199	0.0145987	0.061281	0.1243585	<.0001*
15V	5V	0.0546403	0.0151111	0.021995	0.0872857	0.0031*
15V	10V	0.0499331	0.0145987	0.018395	0.0814716	0.0046*
20V	15V	0.0428869	0.0174488	0.005191	0.0805826	0.0288*
10V	5V	0.0047072	0.0117050	-0.020580	0.0299943	0.6941

(b)

Supplementary Figure S3 : T test for trypan blue experiments (a) Concentration (b) Absorbance

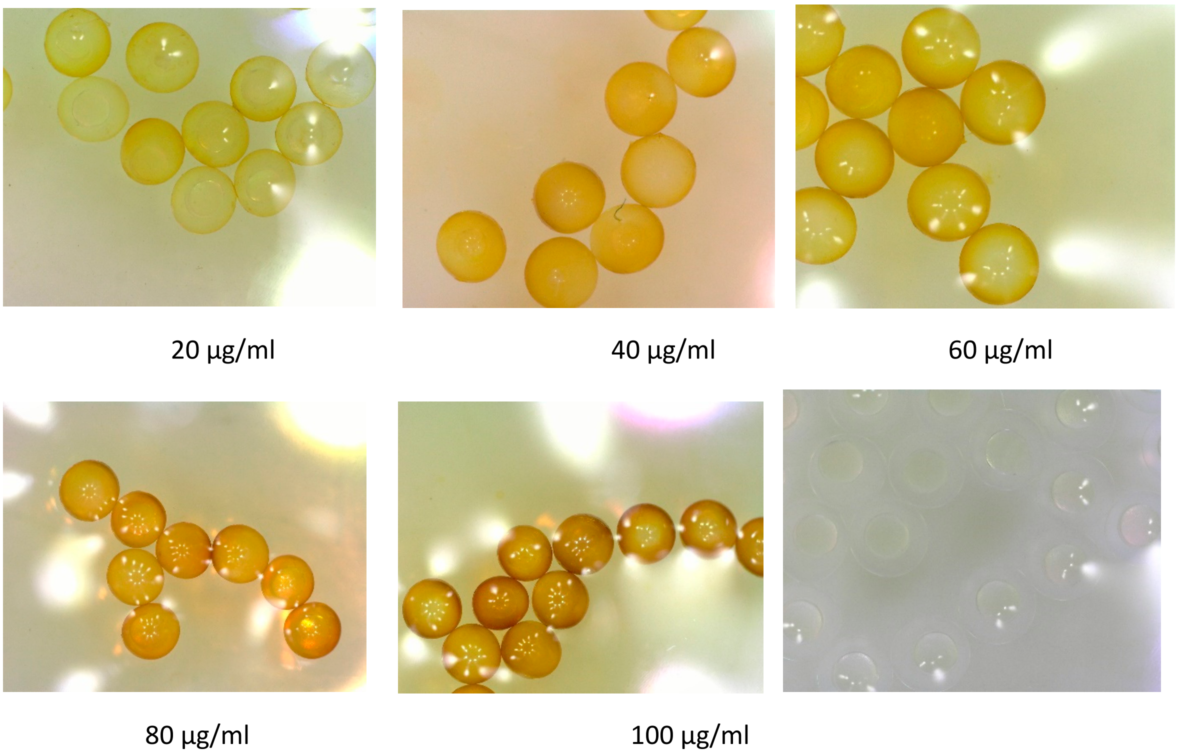

Supplementary Figure S4 : Silver concentration inside chorion after exposure of 5 hours for control experiment

Supplementary Table :

Supplementary Table S1 : Average absorbance for various concentration of trypan blue entered in to the chorion of embryos.

**Trypan blue (mg/ml)**	**Average absorbance**
0	0
0.0004	0.0001
0.004	0.138
0.04	0.53
0.4	0.75

Supplementary Table S2 : Trypan blue concentration and absorbance for various voltage application

Voltage	Concentration (µg/ml)	SD (concentration)	Absorbance	SD (absorbance)
Control	0.913	0.188	0.051	0.023
5V	2.353	0.186	0.166	0.009

10V	2.753	0.220	0.181	0.010
15V	4.034	0.262	0.221	0.036
20V	5.741	0.038	0.264	0.001

Supplementary Table S3 : Survival rate for trypan blue testing

**Voltage**	**No of embryos**	**survived**	**ratio**	**Normalized percentage**
Control	30	20	0.67	100
5V	28	19	0.68	100
10V	29	17	0.59	87.49
15V	23	8	0.35	51.91
20V	20	5	0.25	37.31

Supplementary Table S4 : absorbance calculation for silver and gold nanoparticles.

Sample	Average absorbance	SD	P-value
only AgNP	0.012	0.002	
Voltage+ AgNP	0.042	0.008	0.0017
Only AuNP	0.031	0.001	
Voltage + AuNP	0.041	0.004	0.0059

Supplementary Table S5 : Control experiments results

Concentration(µg/ml)	Absorbance	STD
20	0.16	0.056
40	0.24	0.04
60	0.28	0.058
80	0.31	0.039
100	0.37	0.043

Supplementary Table S6 : Control experiment results

Concentration	Survival percentage	Deformation rate
Control 1	88.23	0
100ug/ml	25	80
80ug/ml	30	75
60ug/ml	37.5	33.33
40ug/ml	50	10
20ug/ml	67.5	0

Supplementary Table S7: No of embryos for different repetition

	Control	only nano	only v	Nano+V
Repeat 1	n=44	n =44	n=25	n=25
Repeat 2	n=50	n=21	n=33	n=30
Repeat 3	n=50	n=36	n=35	n=25
Repeat 4	n=53	n=20	n=28	n=25
Repeat 5	n=51	n=34	n=33	n=25

Supplementary Table S8 : Deformation rate for electroporation experiments

	Day 1	Day 2	Day 3	Day 4	Day 5
Control 1(No V+ No particle)	0	0	0	0	0
Only nano	0	0	20	0	0
Only V	0	0	0	0	3.7
Nano+V	35.71	31.5	50	22	22

Supplementary Table S9: Student t-test for Deformity rate for electroporation experiments

**Condition**	**Average Deformity rate (%)**	**SD**	**P-value**
Control 1(No V+ No particle)	0.00	0.00	-
Only nano	4.00	8.94	0.187
Only V	0.74	1.65	0.187
Nano+V	32.24	11.59	0

Supplementary Table S10 : Heart rate data for electroporation experiments

	Repeat 1(Avg)	Repeat 2(Avg)	Repeat 3(Avg)	Repeat 4(Avg)	Repeat 5(Avg)
Control 1(No V+ NO particle)	107	90	98	109	111
Only nano	112	96	90	98	93
Only V	91	85	102	94	107
Nano+V	98	77	76	74	100

Supplementary Table S11: P-value for students-t test for heart rate data for electroporation experiment

Condition	Repeat 1	Repeat 2	Repeat 3	Repeat 4	Repeat 5

Control 1(No V+ NO particle)	-	-	-	-	-
Only nano	0.2	0.34	0.36	0.47	0.3
Only V	0.16	0.34	0.6	0.92	0.17
Nano+V	0.0008	0.016	0.14	0.012	0.012

Supplementary Table S12: Survival data for electroporation experiments

	Day 1	Day 2	Day 3	Day 4	Day 5
Control 1(No V+ NO particle)	54.83	72	70	50.9	75
Only nano	52.27	71	66.67	50	56
Only V	40	84.8	71.42	53.5	82
Nano+V	56	70	20	36	77
